# Epidemiological Trends in the Timing of In-Hospital Death in Acute Myocardial Infarction-Cardiogenic Shock in the United States

**DOI:** 10.3390/jcm9072094

**Published:** 2020-07-03

**Authors:** Saraschandra Vallabhajosyula, Shannon M. Dunlay, Malcolm R. Bell, P. Elliott Miller, Wisit Cheungpasitporn, Pranathi R. Sundaragiri, Kianoush Kashani, Bernard J. Gersh, Allan S. Jaffe, David R. Holmes, Gregory W. Barsness

**Affiliations:** 1 Department of Cardiovascular Medicine, Mayo Clinic, Rochester, MN 55905, USA; Dunlay.Shannon@mayo.edu (S.M.D.); Bell.Malcolm@mayo.edu (M.R.B.); Gersh.bernard@mayo.edu (B.J.G.); Jaffe.Allan@mayo.edu (A.S.J.); Holmes.David@mayo.edu (D.R.H.); Barsness.Gregory@mayo.edu (G.W.B.); 2 Division of Pulmonary and Critical Care Medicine, Department of Medicine, Mayo Clinic, Rochester, MN 55905, USA; kashani.kianoush@mayo.edu; 3 Center for Clinical and Translational Science, Mayo Clinic Graduate School of Biomedical Sciences, Rochester, MN 55905, USA; 4 Department of Health Science Research, Mayo Clinic, Rochester, MN 55905, USA; 5 Division of Cardiovascular Medicine, Department of Medicine, Yale University School of Medicine, New Haven, CT 06511, USA; Elliott.miller@yale.edu; 6 Division of Nephrology, Department of Medicine, University of Mississippi School of Medicine, Jackson, MS 39216, USA; wcheungpasitporn@gmail.com; 7 Division of Hospital Internal Medicine, Department of Medicine, Mayo Clinic, Rochester, MN 55905, USA; drpranathi99@gmail.com; 8 Division of Nephrology and Hypertension, Department of Medicine, Mayo Clinic, Rochester, MN 55905, USA

**Keywords:** in-hospital death, cardiogenic shock, acute myocardial infarction, cardiac intensive care unit, critical care cardiology, outcomes research

## Abstract

Background: There are limited data on the epidemiology and timing of in-hospital death (IHD) in patients with acute myocardial infarction-cardiogenic shock (AMI-CS). Methods: Adult admissions with AMI-CS with IHDs were identified using the National Inpatient Sample (2000–2016) and were classified as early (≤2 days), mid-term (3–7 days), and late (>7 days). Inter-hospital transfers and those with do-not-resuscitate statuses were excluded. The outcomes of interest included the epidemiology, temporal trends and predictors for IHD timing. Results: IHD was noted in 113,349 AMI-CS admissions (median time to IHD 3 (interquartile range 1–7) days), with early, mid-term and late IHD in 44%, 32% and 24%, respectively. Compared to the mid-term and late groups, the early IHD group had higher rates of ST-segment-elevation AMI-CS (74%, 63%, 60%) and cardiac arrest (37%, 33%, 29%), but lower rates of acute organ failure (68%, 79%, 89%), use of coronary angiography (45%, 56%, 67%), percutaneous coronary intervention (33%, 36%, 42%), and mechanical circulatory support (31%, 39%, 50%) (all *p* < 0.001). There was a temporal increase in the early (adjusted odds ratio (aOR) for 2016 vs. 2000 2.50 (95% confidence interval (CI) 2.22–2.78)) and a decrease in mid-term (aOR 0.75 (95% CI 0.71–0.79)) and late (aOR 0.34 (95% CI 0.31–0.37)) IHD. ST-segment-elevation AMI-CS and cardiac arrest were associated with the increased risk of early IHD, whereas advanced comorbidity and acute organ failure were associated with late IHD. Conclusions: Early IHD after AMI-CS has increased between 2000 and 2016. The populations with early vs. late IHD were systematically different.

## 1. Introduction

Acute myocardial infarction (AMI) continues to be the leading cause of cardiogenic shock (CS) and is associated with poor outcomes [1,2,3,4]. With an increasing emphasis on early recognition, timely percutaneous coronary intervention (PCI) and protocoled AMI-CS care, there have been rapid improvements in the clinical outcomes of this population [5,6,7,8,9]. Several recent studies have detailed the changing demographics, comorbidities and clinical course for patients presenting with AMI-CS [1,2]. It is conceivable that in the last two decades, with early PCI and the use of newer mechanical circulatory support (MCS) devices, the landscape of AMI-CS may have evolved [1,2,5]. Though prior studies have highlighted short-term and long-term mortality in AMI-CS, there are very little published data on the timing of in-hospital death (IHD) in the AMI-CS population [10]. It is also conceivable that the populations with early IHD in AMI-CS may be systematically different from those with late mortality [11]. Therefore, this study sought to investigate the epidemiology of the timing of IHD in patients hospitalized with AMI-CS in the United States. We hypothesized that there would be a decrease in the risk of IHD within two days of admission. We also sought to systematically evaluate the differences in demographics, clinical course and management strategies of patients with IHD early, as compared to later, in hospitalization.

## 2. Material and Methods

The National (Nationwide) Inpatient Sample (NIS) is the largest all-payer database of hospital inpatient stays in the United States. NIS contains discharge data from a 20% stratified sample of community hospitals and is a part of the Healthcare Cost and Utilization Project (HCUP), sponsored by the Agency for Healthcare Research and Quality. Using the HCUP-NIS data from 2000–2016, a retrospective cohort study of admissions with AMI in the primary diagnosis field (International Classification of Diseases 9.0 Clinical Modification (ICD-9CM) 410.x and ICD-10CM I21.x-22.x) and a secondary diagnosis of CS (ICD-9CM 785.51, ICD-10CM R57.0) with IHD were included. Admissions without IHD and length of stay data, those with a do-not-resuscitate status (ICD-9CM V49.86; ICD-10CM Z66.0) and inter-hospital transfers were excluded. Deyo’s modification of the Charlson Comorbidity Index was used to identify the burden of co-morbid diseases (Supplementary Table S1). Demographics, hospital characteristics, acute organ failure, coronary angiography, PCI, MCS and non-cardiac organ support use were identified for all admissions [1,3,4,6,8,12,13,14,15,16,17,18].

The outcomes of interest were the temporal trends of IHD stratified into early (≤2 days), mid-term (3–7 days) and late (>7 days) from 2000–2016. Secondary outcomes included temporal trends stratified by patient and hospital characteristics, predictors of early and late IHD and temporal trends stratified by type of AMI (ST-segment myocardial infarction (STEMI) or non-ST-segment myocardial infarction (NSTEMI)), presence of cardiac arrest and performance of coronary angiography and PCI.

## 3. Statistical Analysis

As recommended by HCUP-NIS, survey procedures using discharge weights provided with the HCUP-NIS database were used to generate national estimates. Using the trend weights provided by the HCUP-NIS, samples from 2000–2011 were re-weighted to adjust for the 2012 HCUP-NIS re-design [19]. One-way analysis of variance and *t*-tests were used to compare categorical and continuous variables, respectively. Logistic regression was used to compare the risk of IHD in each year of the study using the year 2000 as the referent; results are reported as odds ratios (OR) with 95% confidence intervals (CI). A multivariable logistic regression analysis incorporating age, sex, race, primary payer status, socio-economic stratum, hospital characteristics, comorbidities, acute organ failure, AMI-type, cardiac procedures and non-cardiac procedures was performed for temporal trends analyses. For the multivariable modeling, regression analysis with a purposeful selection of statistically (liberal threshold of *p* < 0.20 in univariate analysis) and clinically relevant variables was conducted. Two-tailed *p* < 0.05 was considered statistically significant. All statistical analyses were performed using SPSS v25.0 (IBM Corp., Armonk, NY, USA).

## 4. Results

**Figure 1:**
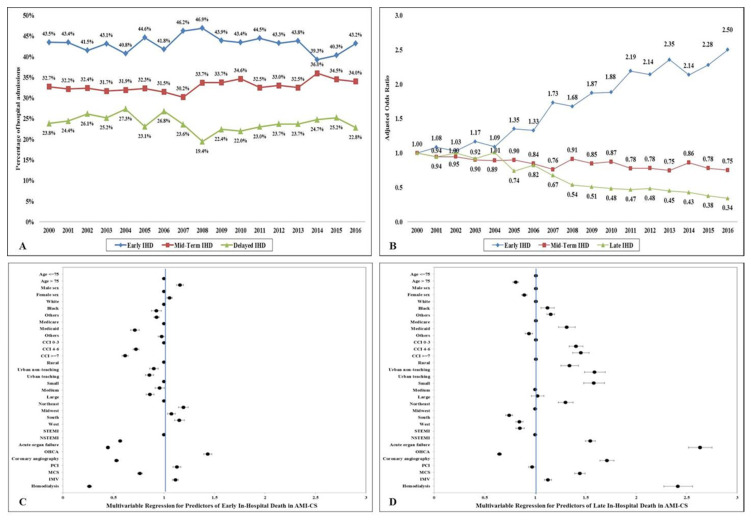
Epidemiology and predictors of in-hospital death in AMI-CS. (**A**) 17-year unadjusted temporal trends of early, mid-term and late IHD in AMI-CS; all *p* < 0.001 for trend over time; (**B**): 17-year adjusted * temporal trends for early, mid-term and late IHD in AMI-CS (referent year 2000); all *p* < 0.001 for trend over time; (**C**,**D**) the adjusted odds ratios for early (**C**) and late (**D**) IHD in AMI-CS **. * Adjusted for age, sex, race, primary payer status, socio-economic stratum, hospital characteristics, comorbidities, acute organ failure, AMI-type, cardiac procedures, and non-cardiac procedures. ** For cohorts with multiple categories (i.e., age, sex, race, primary payer, Charlson Comorbidity Index, hospital location and teaching status, hospital bed size, hospital region and AMI type), the first category was used as reference category for calculating odds ratios. Abbreviations: AMI: acute myocardial infarction; CCI: Charlson Comorbidity Index; CS: cardiogenic shock; IHD: in-hospital death; IMV: invasive mechanical ventilation; MCS: mechanical circulatory support; NSTEMI: non-ST-segment elevation myocardial infarction; OHCA: out-of-hospital cardiac arrest; PCI: percutaneous coronary intervention; STEMI: ST-segment elevation myocardial infarction.

In the period between 1 January 2000 and 31 December 2016, there were 513,288 AMI-CS admissions, of which IHD was noted in 156,366 (38.8%) (Supplementary Figure S1). Of those, 113,349 admissions with a mean length of stay of 5.9 ± 8.9 days (median 3 (interquartile range 1–7) days) met our inclusion criteria and were included in the analysis (Supplementary Figure S1). Early (≤2 days), mid-term (3–7 days) and late (>7 days) IHD were noted in 50,235 (44.3%), 36,227 (32.0%) and 26,886 (23.7%) admissions. The group with early IHD was on average older, more often female, of white race and admitted to small and rural hospitals (Table 1). The 17-year trends showed a temporal increase in early (adjusted OR for 2016 vs. 2000 2.50 (95% CI 2.22–2.78)) and a decrease in mid-term (adjusted OR 0.75 (95% CI 0.71–0.79)) and late (adjusted OR 0.34 (95% CI 0.31–0.37)) IHD (Figure 1A,B).

The cohort with early IHD had higher rates of STEMI-CS and out-of- and in-hospital cardiac arrests, but lower rates of acute organ failure and non-cardiac organ support (Table 2). There was a temporal increase in atrial and ventricular arrhythmias with an increase in timing of IHD. Compared to the admissions with mid-term and late IHD, the cohort with early IHD had a lower use of coronary angiography, PCI, invasive hemodynamic monitoring and MCS (all *p* < 0.001) (Table 2). There was a serial increase in the use of palliative care consultation and in-hospital complications with increasing hospital length of stay.

Admissions with STEMI-CS, concomitant out-of-hospital cardiac arrest and admissions without use of angiography and PCI had consistently higher rates of early IHD during this 17-year study period (Table 1). In a multivariable analysis, older age, white race, admission to a rural hospital, STEMI-CS presentation and out-of-hospital cardiac arrest were independent predictors of early IHD, whereas non-white race, advanced comorbidity, NSTEMI-CS presentation, acute organ failure and non-cardiac organ support were independent predictors of late IHD (Figure 1C,D).

## 5. Discussion

In the first nationally-representative study evaluating the timing of IHD in AMI-CS, early (≤2 days) IHD was noted in nearly half of all AMI-CS admissions, with an increase in prevalence during the 17-year study period. The population with early IHD was systematically different from the other two cohorts—they had lower comorbidity, received less frequent cardiac procedures, had higher rates of STEMI-CS, had higher rates of cardiac arrest, had lower rates of acute non-cardiac organ failure and non-cardiac organ support systems and had lower rates of complications.

The timing of in-hospital events, including IHD remains underexplored in critical illness [1,8,15]. In a nationally-representative population of STEMI admissions that received primary PCI, we previously demonstrated that ventricular arrhythmias were predominant in early in-hospital cardiac arrest, whereas non-shockable rhythms and multi-organ involvement were prevalent in the delayed group [13]. Similarly, Law et al. demonstrated a decline in all time periods of IHD in septic shock patients; however, when adjusted for acute respiratory failure, only the delayed IHD cohort had a temporal decrease [11]. The timing of in-hospital events remains extremely crucial in critical illness since many clinical interventions, such as fluid resuscitation, PCI, MCS, targeted temperature management, vasoactive medications and intensive care monitoring are geared towards the first 24–48 h of critical illness [11,13,16,20]. Therefore, studies evaluating the timing of events aid in prognostication, resource planning and advanced care planning [11,13,16,20]. In AMI-CS, most mortality analyses and predictive models have used IHD, 28-day or 30-day mortality as the end-points, with little additional granularity on the timing of IHD during the index hospitalization [10]. Previous work from our group has demonstrated that AMI-CS patients frequently transition to invasive mechanical ventilation in the first two days, as well as a temporal decrease in the use of prolonged mechanical ventilation and tracheostomy use in the United States [15,20]. Taken in aggregate, these data might suggest that there have been improvements in care delivery for this acutely ill population, decreasing the burden of chronic critical illness [11]. It is possible that patients in the early IHD group may have died before any intervention could be performed, which could potentially explain the lower use of coronary angiography, PCI and MCS in this cohort.

This study has several limitations, some of which are inherent to the analysis of a large administrative database. There is limited information on procedural details, intracoronary anatomy, echocardiographic features (including left ventricular ejection fraction and cardiac index), hemodynamic indices and physiological variables, limiting further patient-specific and disease-specific risk assessment. We are unable to comment on the severity of illness using standardized risk scores and are unable to ascertain the location of admissions within the hospital, which significantly impact these results. Despite these limitations, this study addressed an important knowledge gap, emphasizing the timing of IHD in AMI-CS admissions over the last 17 years.

In conclusion, this nationally-representative 17-year study of AMI-CS admissions noted a temporal increase in early IHD (≤2 days). The populations with early vs. late IHD were systematically different from each other, which suggests that these two populations constitute different phenotypes. Further studies are needed to understand potentially modifiable in-hospital factors that determine the course of AMI-CS with targeted interventions to improve survival in this critically ill population.

## Figures and Tables

**Table 1:** Baseline characteristics of AMI-CS stratified by the timing of IHD.

Characteristic		Early IHD (*n* = 50,235)	Mid-Term IHD (*n* = 36,227)	Late IHD (*n* = 26,886)	*p*
Age (years)		74.2 ± 12.5	73.5 ± 12.3	71.8 ± 11.8	<0.001
Female sex		45.4	43.5	39.1	<0.001
Race	White	63.9	63.6	60.9	<0.001
	Black	6.1	6.5	7.2	
	Others ^a^	30.1	29.9	31.9	
Primary payer	Medicare	73.7	72.6	70.9	<0.001
	Medicaid	4.3	5.3	7.0	
	Private	15.0	15.6	16.6	
	Others ^b^	6.9	6.5	5.5	
Quartile of median household income for zip code	0–25th	22.1	22.2	22.1	<0.001
	26th–50th	26.3	26.7	23.9	
	51st–75th	25.6	24.2	25.4	
	75th–100th	26.0	26.9	28.5	
Hospital teaching status and location	Rural	10.9	8.6	4.9	<0.001
	Urban non-teaching	44.8	44.5	40.9	
	Urban teaching	44.3	47.0	54.2	
Hospital bed size	Small	9.7	8.5	6.9	<0.001
	Medium	25.8	24.7	21.1	
	Large	64.5	66.9	72.0	
Hospital region	Northeast	18.5	18.0	19.5	<0.001
	Midwest	22.4	21.8	20.0	
	South	38.3	40.1	39.0	
	West	20.7	20.1	21.5	
Charlson Comorbidity Index	0–3	17.0	14.4	13.8	<0.001
	4–6	59.7	58.1	60.6	
	≥7	23.4	27.5	25.5	

Legend: Represented as percentage or mean ± standard deviation; ^a^ Hispanic, Asian, Native American, others; ^b^ uninsured, no charge, others. Abbreviations: AMI: acute myocardial infarction; CS: cardiogenic shock; IHD: in-hospital death.

**Table 2:** Clinical characteristics of AMI-CS stratified by the timing of IHD.

Characteristic		Early IHD (*n* = 50,235)	Mid-Term IHD (*n* = 36,227)	Late IHD (*n* = 26,886)	*p*
AMI type	STEMI-CS	73.7	63.1	60.1	<0.001
	NSTEMI-CS	26.3	36.9	39.9	
Acute organ failure	Respiratory	48.0	55.6	65.2	<0.001
	Renal	36.2	51.2	60.0	<0.001
	Hepatic	8.4	13.1	14.3	<0.001
	Hematologic	6.4	12.2	20.3	<0.001
	Neurologic	18.3	21.1	21.7	<0.001
Cardiac arrhythmias	VT	15.2	18.2	22.3	<0.001
	VF	15.7	15.4	13.8	<0.001
	AFib	20.3	27.4	32.9	<0.001
	AFlut	1.3	2.7	6.0	<0.001
	SVT	0.7	1.1	1.6	<0.001
Out-of-hospital cardiac arrest		36.9	32.5	28.8	<0.001
In-hospital cardiac arrest		22.1	15.9	12.5	<0.001
Coronary angiography		45.1	56.2	66.9	<0.001
Percutaneous coronary intervention		32.7	36.3	42.0	<0.001
Coronary artery bypass grafting		3.6	9.3	20.3	<0.001
Invasive hemodynamic monitoring ^a^		13.5	20.0	27.0	<0.001
Mechanical circulatory support	Total	31.1	38.8	50.4	<0.001
	IABP	29.2	37.0	48.3	<0.001
	pLVAD	2.2	2.0	2.2	0.06
	ECMO	0.4	0.7	1.3	<0.001
Invasive mechanical ventilation		53.8	53.0	61.6	<0.001
Non-invasive ventilation		2.2	3.4	4.1	<0.001
Hemodialysis		1.4	5.2	9.7	<0.001
Palliative care consultation		6.2	8.8	8.9	<0.001
Complications	Vascular	0.7	1.2	2.0	<0.001
	Lower limb	0.0	0.1	0.6	<0.001
	VSD	1.3	1.6	1.9	<0.001
	Papillary muscle rupture	0.5	0.3	0.5	<0.001
	Hemopericardium	0.2	0.2	0.5	<0.001
	Cardiac tamponade	0.5	0.6	0.7	0.001
	Coronary dissection	0.6	0.6	0.6	0.79
	Ischemic CVA	1.4	2.6	6.2	<0.001
	Hemorrhagic CVA	0.6	0.8	1.1	<0.001

Legend: Represented as percentage; ^a^ pulmonary artery/right heart catheterization. Abbreviations: AFib: atrial fibrillation; AFlut: atrial flutter; AMI: acute myocardial infarction; CS: cardiogenic shock; CVA: cerebrovascular accident; ECMO: extracorporeal membrane oxygenation; IABP: intra-aortic balloon pump; IHD: in-hospital death; NSTEMI: non-ST-segment elevation myocardial infarction; pLVAD: percutaneous left ventricular assist device; STEMI: ST-segment elevation myocardial infarction; SVT: supraventricular tachycardia; VF: ventricular fibrillation; VSD: ventricular septal defect; VT: ventricular tachycardia.

## Supplementary Materials

The following are available online at https://www.mdpi.com/2077-0383/9/7/2094/s1, Figure S1: Study cohort, Table S1: Administrative codes used for identification of diagnoses and procedures.
