# Supplementary material for: Epidemiological Trends in the Timing of In-Hospital Death in Acute Myocardial Infarction-Cardiogenic Shock in the United States
Source: J Clin Med. 2020 Jul 3;9(7):2094. doi: 10.3390/jcm9072094 (PMC7408956; doi:10.3390/jcm9072094)
Supplement: Supplementary file 1 [file jcm-09-02094-s001.pdf]

SUPPLEMENTARY TABLES

Supplementary Table 1. Administrative codes used for identification of diagnoses and procedures

Comorbidity	International Classification of Diseases, 9.0 Clinical Modifications codes
Cardiac arrest	427.5
Cardiogenic shock	785.51
Acute kidney injury	584, 584.5, 584.6, 584.7, 584.8, 584.9
Respiratory failure	518.81, 518.82, 518.85, 786.09, 799.1, 96.7, 96.70, 96.71, 96.72
Hepatic failure	570.0, 572.2, 573.3, 573.4
Hematologic failure	286.6-286.9, 287.4, 287.5
Neurological failure	293, 293.0, 293.1, 293.8, 293.81-293.84, 293.89, 293.9, 348.1, 348.3, 348.30, 348.81, 348.39, 780.01, 780.09, 89.14
Invasive hemodynamic assessment	37.21, 37.23, 204
Coronary angiography	37.22, 37.23, 88.53-88.56
Percutaneous coronary intervention	00.66, 36.01, 36.02, 36.05, 36.06, 36.07, 88.57
Intra-aortic balloon pump	37.61
Percutaneous mechanical circulatory support	37.68
Extra-corporeal membrane oxygenation	39.65
Invasive mechanical ventilation	96.7, 96.70, 96.71, 96.72
Hemodialysis	39.95

SUPPLEMENTARY FIGURE LEGENDS

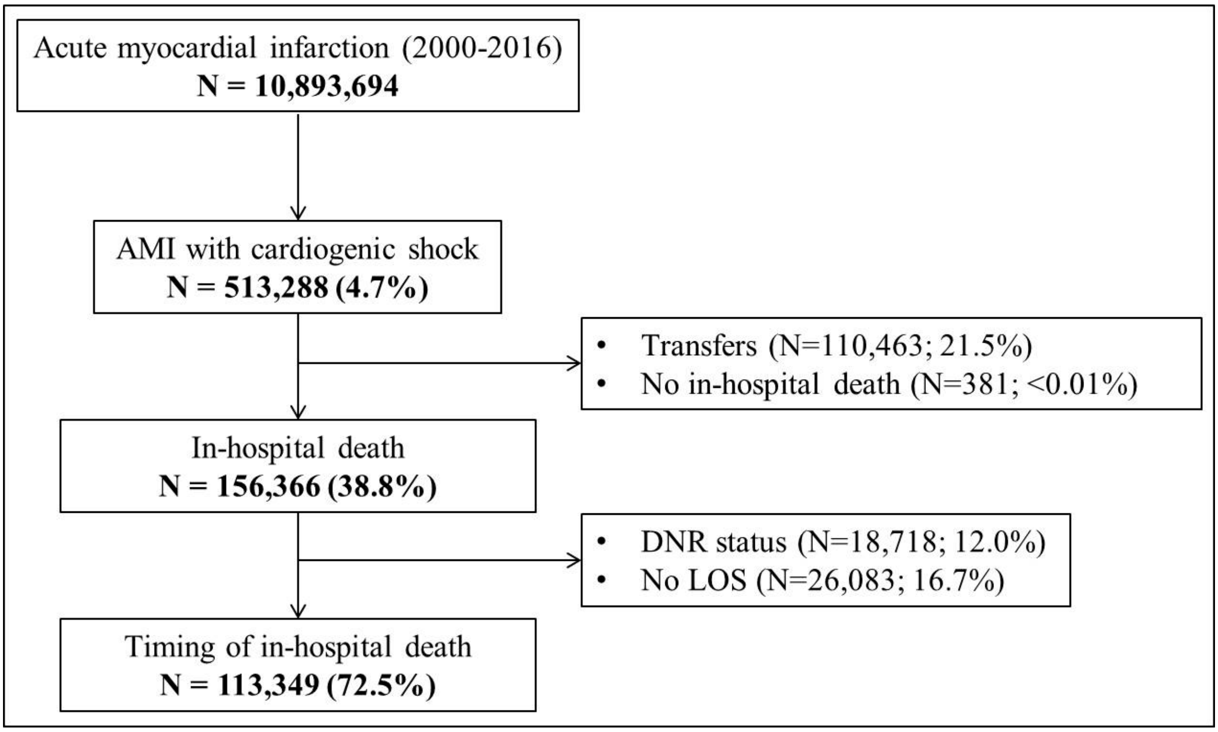

Supplementary Figure 1. Study cohort

Abbreviations: AMI: acute myocardial infarction; DNR: do-not-resuscitate; LOS: length of stay
